# Protein Arginine Methyltransferase 1 (PRMT1) Selective Inhibitor, TC-E 5003, Has Anti-Inflammatory Properties in TLR4 Signaling

**DOI:** 10.3390/ijms21093058

**Published:** 2020-04-26

**Authors:** Eunji Kim, Jiwon Jang, Jae Gwang Park, Kyung-Hee Kim, Keejung Yoon, Byong Chul Yoo, Jae Youl Cho

**Affiliations:** 1 Department of Integrative Biotechnology, Biomedical Institute for Convergence at SKKU (BICS), Sungkyunkwan University, Suwon 16419, Korea; im144069@gmail.com (E.K.); rhea980327@gmail.com (J.J.); keejung@skku.edu (K.Y.); 2 Division of Translational Science, Research Institute, National Cancer Center, Goyang 10408, Korea; wannabejk@naver.com; 3 Proteomic Analysis Team, Research Institute, National Cancer Center, Goyang 10408, Korea; kyunghee@ncc.re.kr

**Keywords:** protein arginine methyltransferase 1 (PRMT1), TC-E 5003, inflammation, AP-1, NF-κB

## Abstract

Protein arginine methyltransferase 1 (PRMT1) is the most predominant PRMT and is type I, meaning it generates monomethylarginine and asymmetric dimethylarginine. PRMT1 has functions in oxidative stress, inflammation and cancers, and modulates diverse diseases; consequently, numerous trials to develop PRMT1 inhibitors have been attempted. One selective PRMT1 inhibitor is *N,N′*-(Sulfonyldi-4,1-phenylene)bis(2-chloroacetamide), also named TC-E 5003 (TC-E). In this study, we investigated whether TC-E regulated inflammatory responses. Nitric oxide (NO) production was evaluated by the Griess assay and the inflammatory gene expression was determined by conducting RT-PCR. Western blot analyzing was carried out for inflammatory signaling exploration. TC-E dramatically reduced lipopolysaccharide (LPS)-induced NO production and the expression of inflammatory genes (inducible NO synthase (iNOS), cyclooxygenase (COX)-2, tumor necrosis factor (TNF)-α and interleukin (IL)-6) as determined using RT-PCR. TC-E downregulated the nuclear translocation of the nuclear factor (NF)-κB subunits p65 and p50 and the activator protein (AP)-1 transcriptional factor c-Jun. Additionally, TC-E directly regulated c-Jun gene expression following LPS treatment. In NF-κB signaling, the activation of IκBα and Src was attenuated by TC-E. Taken together, these data show that TC-E modulates the lipopolysaccharide (LPS)-induced AP-1 and NF-κB signaling pathways and could possibly be further developed as an anti-inflammatory compound.

## 1. Introduction

Inflammation is a biological response against pathogens such as bacteria, fungi and viruses. Immune cells (e.g., macrophages, mast cells, dendritic cells, and neutrophils) recognize damage- or pathogen-associated molecular patterns (DAMPs/PAMPs) through innate immune recognition receptors [1,2]. An encounter between the receptors and pathogens initiates the induction of inflammatory signaling pathways to activate the nuclear factor (NF)-κB, activator protein (AP)-1 and interferon regulatory factor (IRF) via phosphorylation [3,4,5]. In particular, AP-1 activity is commonly regulated by mitogen-activated protein kinases (MAPKs), which are regulated by MAPK kinases (MAPKKs)/transforming growth factor beta-activated kinase 1 (TAK1) [6,7]. The resulting activated transcriptional factors regulate the expression of pro-inflammatory genes including cytokines (interleukin (IL)-1β, tumor necrosis factor (TNF)-α, IL-10), chemokines (eotaxin and macrophage inflammatory protein (MIP)-2) and adhesion molecules (intracellular adhesion molecule (ICAM)-1 and cluster of differentiation (CD29)) to eliminate the pathogen [8,9]. Because dysfunctions of inflammation can cause severe diseases, such as chronic inflammation, arthritis, Alzheimer’s disease and metabolic diseases, inflammatory signaling is tightly controlled [4,10,11].

**Figure 1:**
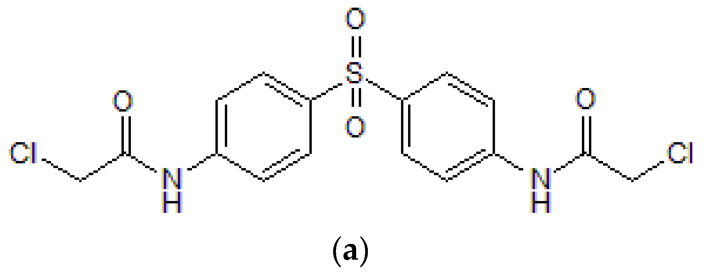

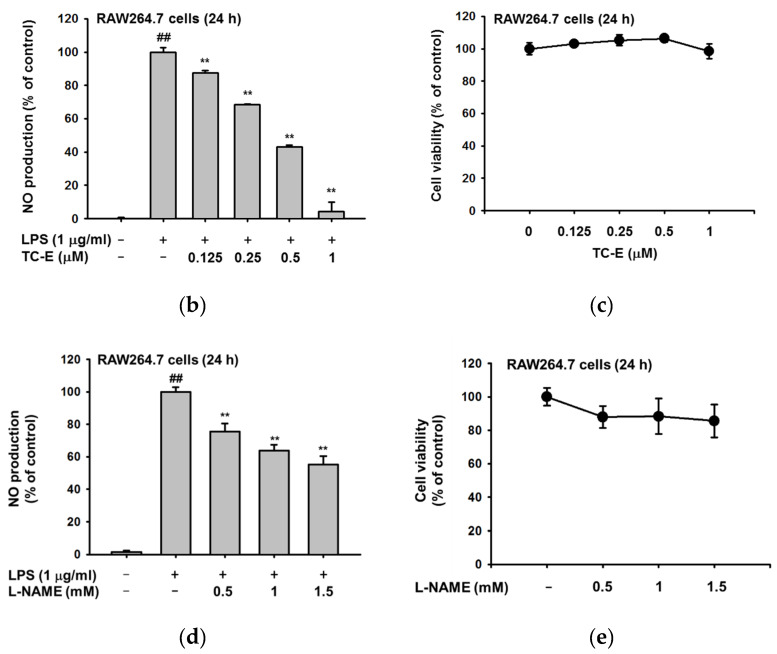
NO production levels in TC-E-treated RAW264.7 cells. (**a**) Structure of *N,N′*-(Sulfonyldi-4,1-phenylene)bis(2-chloroacetamide) or TC-E 5003 (TC-E). RAW264.7 cells were pre-treated with TC-E (0–1 µM) (**b)** or NG-Nitro-l-arginine Methyl Ester (l-NAME) (0–1.5 mM) (**d**) for 30 min, and LPS (1 µg/mL) was added for 24 h. Supernatants were collected, and the production of NO was analyzed using the Griess assay. Optical density at 570 nm was measured using spectrometry. The cytotoxicity of TC-E (0–1 µM) (**c**) and l-NAME (0–1.5 mM) (**e**) were tested using the 3-(4,5-dimethylthiazol-2-yl)-2,5-diphenyltetrazolium bromide (MTT) assay. The data (**b**,**c**,**d**) are expressed as the means ± SEM of the experiments, performed three independent times with five (**b**,**d**) or three (**c**,**e**) samples. All the experiments were performed at least three times with at least three samples. TC-E, TC-E 5003; LPS, lipopolysaccharide; NO, nitric oxide. ^##^ *p* < 0.01 compared to normal group and ^**^ *p* < 0.01 compared LPS-induced group. Statistical significance was analyzed by ANOVA and Kruskal–Wallis test.

Protein arginine methyltransferases (PRMTs) catalyze arginine methylation and add one or two methyl groups from the methyl donor S-adenosylmethionine (AdoMet) [12,13]. The PRMT family is categorized into four types according to the methylarginine derivative generated [14]. All types of PRMTs can generate monomethylated arginine (MMA). Type I PRMTs (PRMT1, PRMT2, PRMT3, PRMT4, PRMT6, PRMT8) also generate asymmetric dimethylarginines (ADMAs), and type II PRMTs (PRMT5 and PRMT9) can form symmetric dimethylarginines (SDMAs) on substrates. PRMT7 is categorized as type III catalyzing only MMA [14,15]. Some type IV methyltransferases catalyze δ-monomethylarginine on internal nitrogen atoms, but this process is not yet fully understood [16]. PRMTs have various substrates including histones and non-histone proteins [17]. Methylated arginines are involved in diverse biological functions such as transcriptional regulation, DNA repair, signal transduction and protein–protein interactions [16]. Among the methyltransferases, PRMT1 is the most prevalent arginine methyltransferase in mammalians and is responsible for various processes including lymphocyte function, oxidative stress, inflammation and cancer [17,18,19,20]. Especially in inflammatory responses, the expression level of PRMT1 was elevated in pulmonary and allergic inflammation [16,21,22]. PRMT1 upregulated the expression of inflammatory genes such as IL-1β, IL-6, CXCL (C-X-C motif chemokine) 10, and CXCL8 [22,23]. Moreover, PRMT1 regulated peroxisome proliferator-activated receptor γ (PPARγ)-dependent macrophage differentiation and major histocompatibility complex II (MHCII) transcription [24,25]. Since PRMT1 is correlated to diseases and is considered a promising target for therapeutic development, several trials have been conducted to discover PRMT1 inhibitors [26,27]. One of the selective PRMT1 inhibitors is the bis-chloroacetyl amide of dapsone (*N,N′*-(Sulfonyldi-4,1-phenylene)bis(2-chloroacetamide), TC-E 5003, Figure 1a). TC-E 5003 (TC-E) shows inhibition solely of PRMT1 and not PRMT4 or the lysine methyltransferase Set7/9 [28]. Based on previous reports that studied the roles of PRMT1 in inflammation [29,30], we investigated the anti-inflammatory roles of TC-E in lipopolysaccharide (LPS)-induced RAW264.7 cells. The production of the inflammatory mediator nitric oxide (NO) and the expression levels of inflammatory genes were measured using Griess assays and RT-PCR, respectively. The activations of the inflammatory AP-1 and NF-κB pathways were verified using Western blotting.

## 2. Results

### 2.1. The Effects of TC-E on NO Production

To determine whether TC-E has anti-inflammatory effects, NO production in RAW264.7 cells was measured after exposure to LPS. TC-E significantly and dose-dependently decreased NO production without cytotoxicity (Figure 1b,c). The reducing effect of TC-E on NO production was analyzed by ANOVA coupled with a Kruskal–Wallis test expressed as mean ± SD. Under the same conditions, the nitric oxide synthase (NOS) inhibitor NG-Nitro-l-arginine Methyl Ester (l-NAME) also suppressed NO production without cytotoxicity (Figure 1d,e). These results showed that TC-E suppressed LPS-induced NO production.

### 2.2. The Effects of TC-E on Pro-Inflammatory Gene Expression

**Figure 2:**
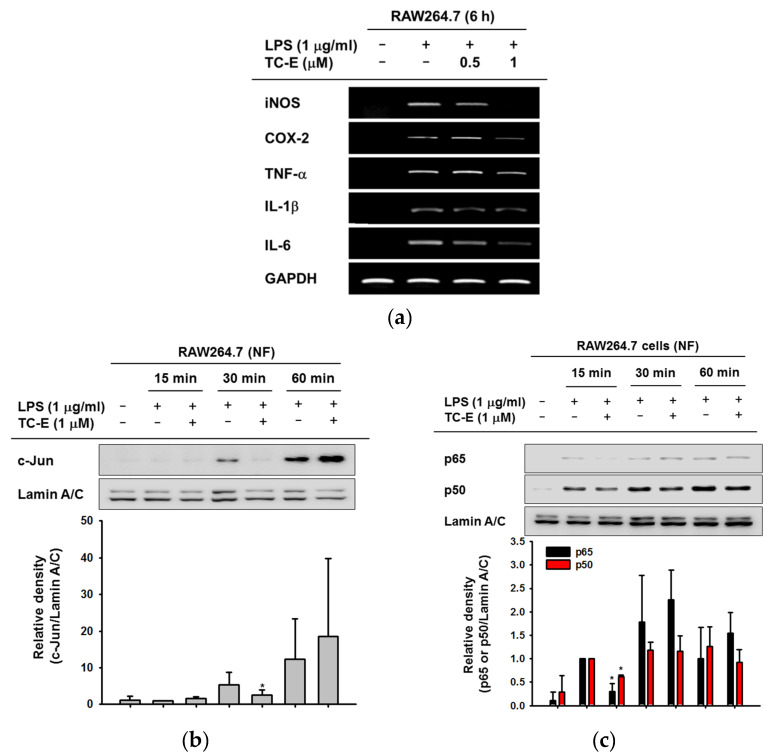
Anti-inflammatory effects of TC-E at the transcriptional level. (**a**) Expression levels of the inflammatory genes in RAW264.7 cells treated with LPS for 6 h. (**b**,**c**) RAW264.7 cells were pre-treated with TC-E then exposed to LPS for various amount of time (0–60 min). Nuclear translocation of the c-Jun subunit of AP-1 (**b**) and the p65 and p60 subunits of NF-κB (**c**) was determined using immunoblotting with Lamin A/C as a standard. All the experiments were performed at least three times with at least three samples. Relative intensity in the bottom panels of b and c is expressed as the mean ± SEM of the data measured and quantified using image J with three different blots of three different samples. iNOS, inducible nitric oxide synthase; COX-2, cyclooxygenase-2; TNF-α, tumor necrosis factor-α; IL-1β, interleukin 1β; IL-6, interleukin 6; GAPDH, glyceraldehyde 3-phosphate dehydrogenase; TC-E, TC-E 5003; LPS, lipopolysaccharide; NF, nuclear fraction. * *p* < 0.05 compared LPS-induced group at each time point. Statistical significance was analyzed by ANOVA and Kruskal–Wallis test.

To confirm that the anti-inflammatory effects of TC-E were the result of transcriptional regulation, the mRNA expression levels of several inflammatory cytokines and mediators were determined using RT-PCR. The expression of inducible NO synthase (iNOS) was remarkably reduced by TC-E and the expressions of COX-2, TNF-α, IL-1β and IL-6 were also downregulated (Figure 2a). Because TC-E downregulated the transcription of inflammatory genes, nuclear proteins were prepared from LPS-treated RAW264.7 cells. After treatment with LPS for 15, 30, or 60 min, the nuclear translocation of the inflammatory AP-1 and NF-κB subunits was determined using immunoblotting. c-Jun, a subunit of the AP-1 transcriptional factor, had less translocation at 30 min following TC-E treatment (Figure 2b). In the case of NF-κB, the translocation of p65 and p50 into the nucleus was suppressed at 15 min of the LPS treatment (Figure 2c). Taken together, these data indicate that TC-E has the ability to regulate inflammatory responses through transcription inhibition. 

### 2.3. The Regulatory Effects of TC-E on the AP-1 Signaling Pathway

**Figure 3:**
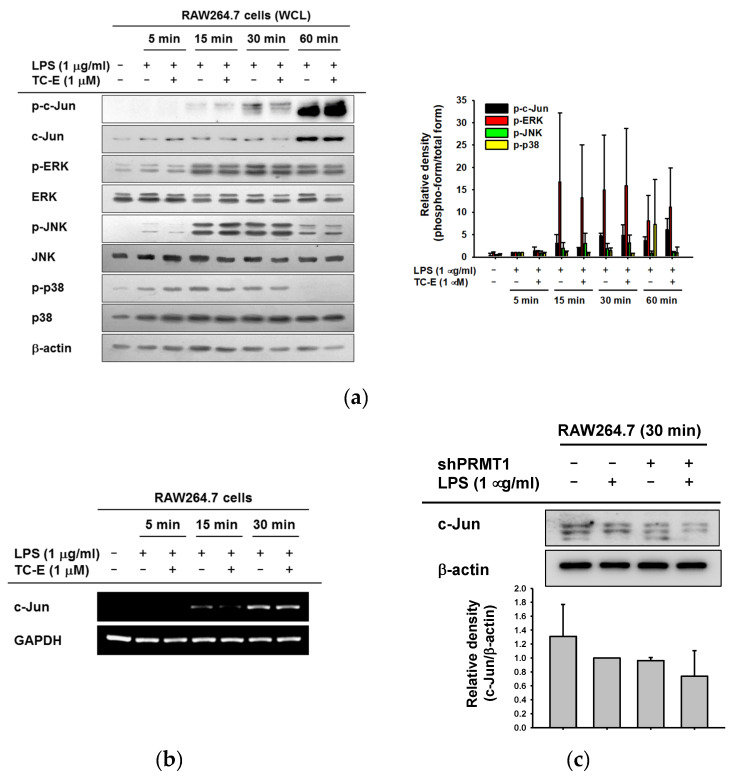
Regulatory mechanism of TC-E on activator protein (AP)-1 activity. (**a**) RAW264.7 cells were pre-treated with TC-E for 30 min then treated with LPS for various amounts of time. Whole cell lysates were harvested and used for immunoblotting with the phospho- and total antibodies against c-Jun, ERK, JNK and p38 with β-actin as a standard. (**b**) The gene expression level of c-Jun was evaluated in the presence or absence of TC-E in RAW264.7 cells treated with LPS for various amounts of time. (**c**) Scrambled- or PRMT1-knockdown RAW264.7 cells were exposed to LPS (1 µg/mL) for 30 min. Whole cell lysates were then collected, and the expression level of c-Jun was determined using immunoblotting. Relative intensity in the right panel of a and the bottom panel of c is expressed as means ± SEM of the data measured and quantified using image J with the three different blots of three different samples. Statistical significance was analyzed by ANOVA and Kruskal–Wallis test. ERK, extracellular signal-regulated kinase; JNK, c-Jun N-terminal kinase; GAPDH, glyceraldehyde 3-phosphate dehydrogenase; PRMT1, protein arginine methyltransferase 1; TC-E, TC-E 5003; LPS, lipopolysaccharide; WCL, whole cell lysates.

Based on previous results (Figure 2b), we determined the regulatory mechanism of TC-E in the inflammatory AP-1 signaling pathway. MAPKs are phosphorylated and activated by LPS, and the signal is transduced to AP-1 subunits through phosphorylation [6,31,32,33]. We examined the phosphorylation levels of c-Jun and MAPKs in the LPS-treated RAW264.7 cells lysates (Figure 3a). The levels of p-c-Jun were decreased by TC-E at 15 and 30 min of exposure to LPS. However, the phosphorylated forms of the MAPKs were not affected by TC-E at any time point. This indicated that TC-E exclusively modulated the amount of nuclear c-Jun but did not affect the phosphorylated forms of MAPKs. Moreover, TC-E suppressed the c-Jun transcription in RAW264.7 cells after 15 min of LPS treatment (Figure 3b). To confirm the inhibitory effect of TC-E on c-Jun, the c-Jun expression levels were determined under PRMT1-knockdown conditions (Figure 3c). The total c-Jun expression was clearly reduced in short hairpin RNA to PRMT1 (shPRMT1)-expressing RAW264.7 cells by LPS exposure. Taken together, these data showed that TC-E regulates LPS-induced AP-1 transcriptional activity by modulating the c-Jun gene expression.

### 2.4. The Regulatory Effects of TC-E on the NF-κB Signaling Pathway 

**Figure 4:**
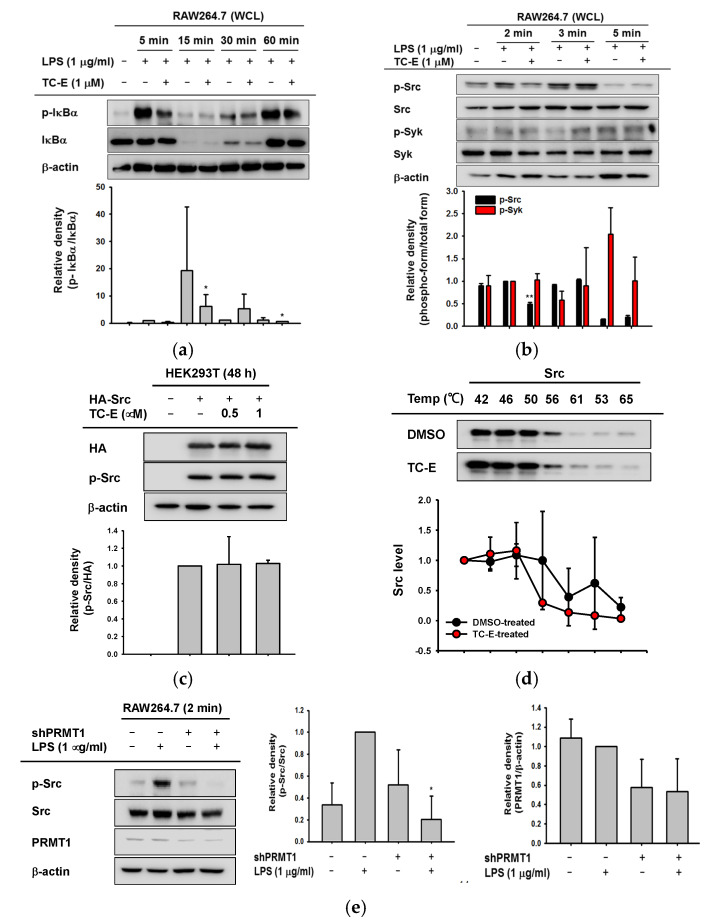
Regulatory mechanism of TC-E on NF-κB activity. (**a**) TC-E-treated RAW264.7 cells were exposed to LPS for various amounts of time (0–60 min), and the whole cell lysates were used to determine IκBα phosphorylation. (**b**) The anti-inflammatory effects of TC-E were also determined in the RAW264.7 cells treated with LPS for shorter amounts of time (0–5 min). The activities of Src and Syk were determined using immunoblotting. (**c**) human embryonic kidney 293T (HEK293T) cells were transfected with HA-Src for 24 h and then treated with TC-E for an additional 24 h. The cells were then harvested, lysed and subjected to immunoblotting using the antibodies against HA and the phosphorylated Src with β-actin as a standard. (**d**) HEK293T cells overexpressing HA-Src were incubated for 24 h in the presence or absence of TC-E (1 µM). The cells were collected with phosphate-buffered saline (PBS) and aliquoted into 0.2-mL tubes containing the same number of cells. The samples were heated at temperatures ranging from 42 to 65 °C, and the levels of Src were determined using immunoblotting. (**e**) Scrambled- or shPRMT1-expressing RAW264.7 cells were treated with LPS (1 µg/mL) for 2 min. Whole cell lysates were used for immunoblotting with the antibodies against phospho-Src, total Src and PRMT1 with β-actin as a standard. All the experiments were performed at least three times. Relative intensity in the bottom panels of (**a**,**c**,**d**), the middle panel of e, and the right panels of b and e is expressed as means ± SEM of the data measured and quantified using image J with the three different blots of three different samples. Statistical significance was analyzed by ANOVA and a Kruskal–Wallis test. * *p* < 0.05 compared to control groups treated with LPS alone at each time point or vehicle control. DMSO, dimethyl sulfoxide; PRMT1, protein arginine methyltransferase 1; TC-E, TC-E 5003; LPS, lipopolysaccharide; WCL, whole cell lysates.

NF-κB signaling was also explored to verify the regulatory mechanism of TC-E. Phosphorylated IκBα was significantly downregulated by TC-E after 5 min of LPS treatment (Figure 4a). Therefore, we ascertained the activation of Src and Syk kinases, which are upstream molecules of the NF-κB signaling pathway, at earlier time points. RAW264.7 cells were treated with LPS for 2, 3, or 5 min, and the activated levels of Src and Syk kinases were observed. (Figure 4b). Although the phosphorylation of both the Src and Syk kinases was activated by LPS, only Src phosphorylation was inhibited by TC-E after 2 min of LPS treatment. Based on these data, we provisionally hypothesized that the Src kinase was a target of TC-E and conducted Src-overexpression experiments and cellular thermal shift assays (CETSAs) using human embryonic kidney 293T (HEK293T) cells. To investigate the role of TC-E on the expected target protein, Src kinase, we set up the Src-overexpressed HEK293T cells to focus on the interested protein because RAW264.7 cells were known to be fastidious for transfection [34,35]. However, the phosphorylation of Src was not altered by TC-E (Figure 4c), and there was no difference in Src stability with or without TC-E (Figure 4d). These results indicated that TC-E modulated Src activity but not in a direct manner. We also confirmed that the inhibitory effects of TC-E were mediated through the PRMT1 regulation. As shown in Figure 4e, Src was not activated by LPS in the PRMT1-knockdown cells but was activated in scrambled-knockdown cells. Taken together, these data show that TC-E exerts an anti-inflammatory effect by controlling the NF-κB signaling pathway.

## 3. Discussion

**Figure 5:**
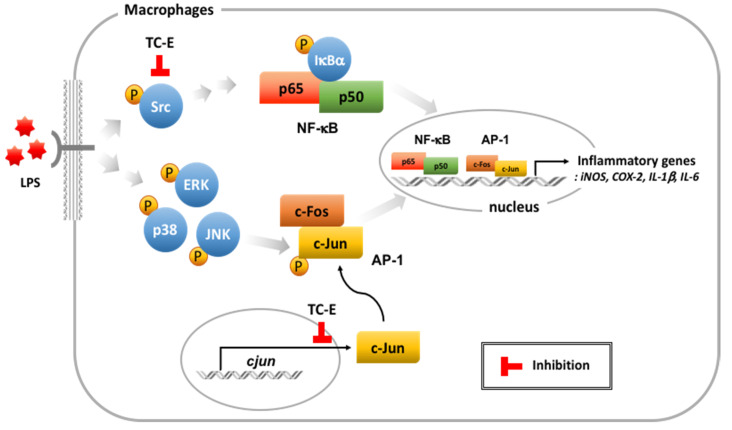
Inhibitory pathway of TC-E in the LPS-induced inflammatory responses. TC-E suppresses the NF-κB signaling by regulating the Src activation. Gene expression of c-Jun is inhibited by TC-E treatment under the LPS-mediated inflammatory conditions. NF-κB, nuclear factor κB; AP-1, activator factor 1; ERK, extracellular signal-regulated kinase; JNK, c-Jun N-terminal kinase; iNOS, inducible nitric oxide synthase; COX-2, cyclooxygeanse-2; IL-1β, interleukin 1β; IL-6, interleukin 6; TC-E, TC-E 5003; LPS, lipopolysaccharide.

We explored the anti-inflammatory effects of the PRMT1 inhibitor TC-E under LPS induction. TC-E clearly diminished the LPS-mediated NO production (Figure 1) and the transcription of the inflammatory gene was also regulated by TC-E (Figure 2). TC-E modulated the AP-1 transcriptional factor activation by suppressing the c-Jun expression (Figure 3). NF-κB signaling was also downregulated by suppressing the activation of Src kinases (Figure 4). These data show that TC-E exerts anti-inflammatory effects by controlling inflammatory molecules in diverse ways (Figure 5). 

TC-E regulated the c-Jun activity at the transcriptional level (Figure 3b) leading to less activation of downstream inflammatory genes such as iNOS, COX-2 and IL-6. Confirming with PRMT1 knock-down cells, there was a much lower level of total c-Jun. Since PRMT1 dimethylates histone 4 at arginine 3 (H4R3), gene expression can be altered by PRMT1. PPARγ is a key transcriptional factor in M2 macrophage polarization for retaining anti-inflammatory states [36,37]. PRMT1 dimethylates the H4R3 associated with the PPARγ gene resulting in the downregulation of the PPARγ response to the IL-4 treatment [37]. In the case of hypoxia, PRMT1 represses the recruiting of both specificity protein 1 (Sp1) and Sp3 transcriptional factors on the hypoxia-inducible transcription factor (HIF)-1α promoter sites [38]. As these previous reports have shown, it is possible that PRMT1 regulates the inflammatory AP-1 transcriptional activity by suppressing the c-Jun subunit availability under early LPS-induced inflammatory conditions. Moreover, we suggested a new regulatory mechanism of c-Jun by PRMT1. Although there are few reports regarding the correlation of c-Jun or other AP-1 transcription factors with PRMT1, one study showed that PRMT1-mediated arginine methylated c-Jun coactivator RING domain AP-1 co-activator-1 (RACO-1) is required for the c-Jun/AP-1 activation [39]. It remains necessary to accurately determine the mechanism of PRMT1 regarding c-Jun/AP-1 activity, but this study demonstrated that PRMT1 activated the LPS-induced inflammatory responses and that TC-E might directly target c-Jun transcriptional activity.

In NF-κB signaling, the phosphorylation of Src was clearly decreased by TC-E after LPS exposure for 2 min (Figure 2b). Src is considered an upstream molecule in NF-κB signaling and is directly regulated by various anti-inflammatory reagents [1,40,41]. However, in this study, TC-E did not directly bind to or control Src kinase activity (Figure 4c,d). Src can be activated by protein kinase C (PKC) γ or be regulated by protein tyrosine phosphatase (PTP) or C-terminal Src kinase (CSK) [42,43]. In particular, PTPs dephosphorylate tyrosine 527 (Y527) of Src, which is an auto-inhibitory residue, causes a conformation change [42,44]. In toll-like receptor 4 (TLR4) signaling, the activity of Src kinases is also regulated by receptor tyrosine kinases (RTKs) such as human epidermal growth factor receptor 2 (HER2), platelet-derived growth factor receptor, [43,45] or TNF receptor-associated factor 6 (TRAF6) [46]. It is possible that TC-E regulates other factors to control the TLR4-mediated Src activation in an indirect way. In our previous report, a plant extract suppressed Src activity, not by direct targeting, but via TLR4/MyD88 signal transduction [47]. 

The trials to develop PRMT inhibitors have been processed because PRMT is one of the prime targets for diverse diseases. AMI-1 (7,7′-carbonylbis(azanediyl)bis(4-hydroxynaphthalene-2-sulfonic acid), a pan PRMT inhibitor, was widely used for studying the biological functions of PRMT1, e.g., pulmonary inflammation, multidrug resistance or RNA processing [48,49]. However, there is a limit to define the accurate roles of PRMT1 by treating this inhibitor, so selective PRMT1 inhibitors were developed and utilized, including furamidine, allantodapsone or MS023 [50,51,52]. TC-E was one of the chemicals developed to specifically inhibit PRMT1 activity and has been used to study the role of PRMT1 in myogenesis and hyperglycemia [53,54]. The inhibitory activity of TC-E in inflammatory responses has not yet been completely understood. In this study, we proved that the TLR4-mediated inflammatory c-Jun (Figure 2b) and NF-κB (Figure 2c) activation was suppressed by treatment with TC-E. It has been widely reported that PRMT1 upregulates inflammatory genes and is involved in inflammatory responses [30,48,55]. Overall, this study suggested that PRMT1 is a potential target for treating inflammatory diseases and that TC-E specifically could be suitable as an anti-inflammatory drug. 

In conclusion, TC-E regulates inflammatory responses by inhibiting the c-Jun expression and NF-κB signal transduction. By downregulating these transcriptional factors, the expression of various inflammatory genes is also inhibited. Collectively, these results show that TC-E has potential for development as an anti-inflammatory compound that targets Src or c-Jun activation.

## 4. Materials and Methods

### 4.1. Materials

TC-E 5003 was purchased from Tocris Bioscience (Bristol, UK). RAW264.7 cells, concatenation of Bagg and Albino (BALB/c)-derived murine macrophage cell line (No. TIB-71), and HEK293T cells and a human embryonic kidney cell line (No. CRL-3216) were acquired from American Type Culture Collection (ATCC) (Rockville, MD, USA). Lipopolysaccharide (LPS, Escherichia coli 0111:B4), l-NAME (NG-Nitro-l-arginine Methyl Ester), puromycin and polyethylenimine (PEI) were obtained from Sigma (St. Louis, MO, USA). 3-(4,5-dimethylthiazol-2-yl)-2,5-diphenyltetrazolium bromide (MTT) was purchased from Amresco (Solon, OH, USA). Fetal bovine serum (FBS) was purchased from Biotechnics Research (Lake Forest, CA, USA), and Roswell Park Memorial Institute 1640 (RPMI1640) and Dulbecco’s Modified Eagle Medium (DMEM) were obtained from HyClone (Grand Island, NY, USA). Antibodies against the total or phosphorylated forms of c-Jun, p65, p50, IκBα, Src, Syk, p38, extracellular signal-regulated kinase (ERK), Lamin A/C, and β-actin were purchased from Cell Signaling (Beverly, MA, USA). The antibodies against the total and phosphorylated forms of c-Fos, c-Jun N-terminal kinase (JNK) and HA were acquired from Santa Cruz Biotechnology Inc. (Dallas, TX, USA). 

### 4.2. Cell Culture

RAW264.7 cells were cultured in an RPMI1640 medium containing 10% heat-inactivated FBS and 1% penicillin-streptomycin. HEK293T cells were incubated in DMEM supplemented with 5% heat-inactivated FBS and 1% penicillin-streptomycin. All the cells were maintained in a 5% CO_2_ humidified incubator at 37 °C.

### 4.3. NO Production and Griess Assay

RAW264.7 cells (1 × 10^6^ cells/mL) were plated in 96-well plates and incubated overnight. The cells were pre-treated with TC-E (0-1 µM) or l-NAME (0–1.5 mM) for 30 min, and then LPS (1 µg/mL) was added. The cells were incubated for an additional 24 h, and the supernatants of the cells were collected to determine the levels of NO production using the Griess assay as previously reported [56,57]. 

### 4.4. Cell Viability Assay

RAW264.7 cells (1 × 10^6^ cells/mL) or HEK293T cells (5 × 10^5^ cells/mL) were plated in 96-well plates and treated with TC-E or l-NAME at various concentrations for 24 h. The conventional MTT assays were then performed as previously described [58,59]. 

### 4.5. Preparation of mRNA and Semiquantitative PCR

RAW264.7 cells (1 × 10^6^ cells/well) were plated on 12-well plates and incubated overnight. TC-E-pre-treated cells were exposed to LPS for the indicated time points. The medium was discarded, and the total RNA was isolated from the cells with TRIzol reagent following the manufacturer’s instructions. Using these mRNA, reverse transcription PCR was performed as previously described [60]. Briefly explained, complementary DNA (cDNA) was synthesized using RevertAid First Strand cDNA synthesis kit (Thermo Fisher Scientific, Waltham, MA, USA). The synthesized cDNA was used for amplifying the target genes with specific primers and the PCRBIO HS Taq PreMix (PCR Biosystems, London, United Kingdom) in thermal cycler (Life Technologies, Carlsbad, CA, USA). The PCR reaction was conducted with the incubation mixture (2 µL cDNA, 4 µM 5′ and 3′ primers, a 10× buffer (10 mM of Tris-Hydrogen chloride (HCl), pH 8.3, 50 mM of KCl, 0.1 % Triton X-100), 250 µM of dNTP, 25 mM of MgCl_2_, and 1 unit of Taq) under the following incubation conditions (a 45s denaturation time at 94 °C, an annealing time of 45 s between 55 to 60 °C, an extension time of 60 s at 72 °C, and a final extension of 7 min at 72 °C at the end of 30 cycles). The primers (Bioneer, Seoul, Korea) used in this experiment are indicated in Table 1 (F: forward, R: reverse). 

### 4.6. Plasmid Transfection 

HEK293T cells (3 × 10^5^ cells/mL) were seeded in 12-well plates to make 70%–80% cell confluency. The cells were then transfected with HA-Src plasmids using PEI for 24 h [61], and then were treated with TC-E for an additional 24 h. After the HEK293T cells were harvested and utilized for analyzing proteins or CETSA.

### 4.7. Preparation of Nuclear Fraction

The cells were harvested with PBS and centrifuged (12,000 rpm for 1 min) once. PBS was removed, and 400 µL of Buffer A (10 mM HEPES, pH 7.8 with KOH; 10 mM KCl; 2 mM MgCl_2_; 0.1 mM ethylenediaminetetraacetic acid (EDTA); 1 mM DTT; 0.1 mM PMSF; 2 µg/mL leupeptin; 2 µg/mL aprotinin) was added for washing and then discarded. The cells were added with Buffer A again with 25 mL 10% NP-40, and vortexted vigorously for 30 s. The cells were centrifuged at 14,000 rpm for 30 s, and the supernatants were carefully discarded. Pellets (nuclear fraction) were washed with Buffer A and suspended with Buffer B (10 mM HEPES, pH 7.8 with KOH; 50 mM KCl; 300 mM NaCl; 0.1 mM EDTA; 1 mM DTT; 0.1 mM PMSF; 2 µg/mL leupeptin; 2 µg/mL aprotinin; 10% (*v*/*v*) glycerol). The nuclear proteins were incubated at 4 °C for 20 min vortexing every 5 min and stored at –80 °C until use.

### 4.8. Preparation of Whole Cell Lysates

The cells were washed with PBS once, collected, transferred to fresh tubes and centrifuged at 12,000 rpm for 5 min at 4 °C. The cells were lysed with lysis buffer (20 mM Tris-HCl, pH 7.4; 2 mM EDTA; 2 mM ethyleneglycotetraacetic acid (EGTA); 1 mM DTT; 50 mM β-glycerol phosphate; 0.1 mM sodium vanadate; 1.6 mM pervanadate; 1% Triton X-100; 10% glycerol; 10 µg/mL aprotinin; 10 µg/mL pepstatin; 1 µM benzamide; and 2 µM PMSF). The protein lysates were then pelleted using centrifugation at 12,000 rpm for 5 min at 4 °C. The resulting supernatants were used for Western blotting.

### 4.9. Western Blotting Analysis

The total cell lysates or nuclear fraction were prepared from RAW264.7 cells or HEK293T cells under the indicated conditions. The protein samples were separated by sodium dodecyl sulfate-polyacrylamide gel electrophoresis (Bio-Rad, Hercules, CA, USA), and immunoblotting was performed with specific antibodies, as reported previously [62]. The nuclear fractions were analyzed with antibodies against the phosphorylated or total forms of c-Jun, p65, p50, and Lamin A/C. With whole cell lysates, the protein levels were examined with antibodies against the phosphorylated or total forms of c-Jun, ERK, JNK, p38, IκBα, Src, Syk, PRMT1, HA, and β-actin. The band intensity in each blot was measured and quantified using image J (Ver. 2) (the National Institutes of Health and the Laboratory for Optical and Computational Instrumentation (LOCI, University of Wisconsin, Madison, WI, USA).

### 4.10. Lentivirus-Mediated Knockdown (shRNA)

Plasmids containing short hairpin RNA (shRNA) coding sequences against either mouse *prmt1* or a non-targeting scrambled sequence (Table 2) were cloned into the pLKO.1 puro vector (a gift from Prof. Yoon, Sungkyunkwan University, Suwon, Republic of Korea) according to the Addgene protocol (www.addgene.org). The lentivirus was then produced by the transient transfection of the HEK293T cells. The generated viruses were used to infect the RAW264.7 cells, and the cells infected with either shScrambled or shPRMT1 were selected using puromycin (2.5 µg/mL) treatment. The knockdown level of the selected cells was confirmed using immunoblotting. 

### 4.11. Statical Analysis

Since our experimental sample scale in this study is not enough to follow up the normal distribution pattern, all the results were analyzed by a non-parametric test using analysis of variance (ANOVA) coupled with the Kruskal–Wallis test. *p*-values < 0.05 were considered statistically significant. The data (Figure 1b–d) presented in this paper are expressed as the means ± SEM of the experiments, performed three independent times with five (Figure 1b,d) or three (Figure 1c,e) samples. The data (bottom and right panels of Figure 2b,c, Figure 3a,c, and Figure 4a–e) are expressed as the mean ± SEM of the relative band intensity values, obtained by Image J, with three different blots of independently prepared three samples. Similar experimental cellular and immunoblotting data were also observed using an additional independent set of experiments that was conducted using the same numbers of experimental samples. All of the statistical tests were carried out using the computer program SPSS (Ver. 24, SPSS Inc., Chicago, IL, USA).

## Figures and Tables

**Table 1:** Sequences of the primers of the investigated genes in a RT-PCR analysis.

Gene		Primer Sequences
*TNF-α*	F	5’-TTGACCTCAGCGCTGAGTTG-3’
	R	5’-CCTGTAGCCCACGTCGTAGC-3’
*IL-1β*	F	5’-CAGGATGAGGACATGAGCACC-3’
	R	5’-CTCTGCAGACTCAAACTCCAC-3’
*IL-6*	F	5’-GTACTCCAGAAGACCAGAGG-3’
	R	5’-TGCTGGTGACAACCACGGCC-3’
*iNOS*	F	5‘-CCCTTCCGAAGTTTCTGGCAGCAGC-3`
	R	5‘-GGCTGTCAGAGCCTCGTGGCTTTGG-3`
*COX-2*	F	5′-CACTACATCCTGACCCACTT-3′
	R	5′-ATGCTCCTGCTTGAGTATGT-3′
*c-Jun*	F	5′- ACGACCTTCTACGACGATGC-3′
	R	5′- CCAGGTTCAAGGTCATGCTC-3′
*GAPDH*	F	5’-CACTCACGGCAAATTCAACGGCAC-3’
	R	5’-GACTCCACGACATACTCAGCAC-3’

**Table 2:** The primers for short hairpin RNA (shRNA) sequences

Gene	Sequences
*shScrambled*	TCCTAAGGTTAAGTCGCCCTCG
*shPRMT1*	CATGATGCAGTTCGCGGCCTCGG

## Abbreviations

ADMA	Asymmetric dimethylarginine
AdoMet	S-Adenosylmethionine
AP-1	Activator protein 1
CETSA	Cellular thermal shift assay
CSK	C-terminal Src kinase
DAMPs	Damage-associated molecular patterns
EDTA	Ethylenediaminetetraacetic acid
EGTA	Ethyleneglycotetraacetic acid
ERK	Extracellular signal-regulated kinase
HIF	Hypoxia-inducible transcription factor
ICAM	Intracellular adhesion molecule
IL	Interleukin
IRF	Interferon regulatory factor
JNK	c-Jun N-terminal kinase
MAPKs	Mitogen-activated protein kinases
MIP	Macrophage inflammatory protein
MMA	Monomethylated arginine
NF-kB	Nuclear factor kB
NO	Nitric oxide
NOS	Nitric oxide synthase
PAMPs	Pathogen-associated molecular patterns
PKC	Protein kinase C
PPAR	Peroxisome proliferator-activator receptor
PRMT	Protein arginine methyltransferase
PTP	Protein tyrosine phosphatase
RTK	Receptor tyrosine kinase
SDMA	Symmetric dimethylarginine
Sp1	Specificity protein 1
TNF	Tumor necrosis factor
TRAF	TNF receptor associated factor
